# Effect of IL-7 and IL-15 on T cell phenotype in myelodysplastic syndromes

**DOI:** 10.18632/oncotarget.8459

**Published:** 2016-03-29

**Authors:** Wen Dong, Tingting Ding, Lei Wu, Xiubao Ren, P.K. Epling-Burnette, Lili Yang

**Affiliations:** ^1^ Department of Orthopaedic Surgery, Tianjin Hongqiao Hospital, Tianjin, P.R. China; ^2^ Department of Immunology, Tianjin Cancer Institute and Hospital, Tianjin Medical University, P.R. China; ^3^ National Clinical Research Center of Cancer, P.R. China; ^4^ Tianjin Key Laboratory of Cancer Immunology and Biotherapy, Tianjin, P.R. China; ^5^ Immunology Program at the H. Lee Moffitt Cancer Center, Tampa, FL, USA

**Keywords:** myelodysplastic syndromes, T cells, IL-15, IL-7

## Abstract

Aberrant T cell phenotype is one of the characteristics of myelodysplastic syndromes (MDS). In this study, we detected an increased concentration of IL-15 in the plasma of MDS patients (*n* = 20) compared with that in the plasma of healthy controls (*n* = 20). In MDS patients, reduced naïve CD4+ and CD8+ T cells [16.11 ± 6.56 vs. 24.11 ± 7.18 for CD4+ T cells (p < 0.001) and 13.15 ± 5.67 vs. 23.51 ± 6.25 for CD8+ T cells (p < 0.001)] were observed. The reduced naïve and increased effector memory T cells were significantly correlated with IL-15 plasma level. Then, the effect of IL-15 and IL-7 was tested *in vitro*. Peripheral blood mononuclear cells from MDS were treated for 15 days with IL-15. This treatment significantly decreased naïve CD4+ (p < 0.001) and CD8+ (p < 0.001) T cells and correspondingly increased terminal memory CD4+ and CD8+ T cells (p < 0.001). Treatment with IL-7 increased naïve CD4+ (p < 0.05) and CD8+ (p < 0.001) T cells. Our results indicated that exposure to high levels of IL-15 may be involved in the T cell phenotype conversion observed in MDS. IL-7 may be one of the promising therapeutic candidates for recovering the effector immune compartment in MDS patients.

## INTRODUCTION

Myelodysplastic syndromes (MDS) include a spectrum of age-related hematological neoplasms characterized by dysplasia, cytopenias, and potential for acute myeloid leukemia (AML) progression [1]. In MDS, T cells are characterized by skewed repertoire distribution [2] and form lymphoid aggregates within the bone marrow of a few patients [3, 4]. Many studies have investigated the role of the immune system in MDS pathogenesis, such as T cell receptor V skewing [5], reduced CD4/CD8 ratio, decreased natural killer (NK) cell function [6], contracted T cell repertoire, and loss of naïve T cells [7]. These findings provide important insight into the disease pathogenesis. However, mechanisms contributing to the development of dysfunctional cellular immunity in MDS patients are unknown.

Several MDS patients progress to acute AML. The suppressive bone marrow microenvironment plays a pivotal role in cancer initiation and progression. In addition, aberrant cytokine expression is present in lymphoid and myeloid cells and is believed to contribute to the disease phenotype and outcome of patients [8, 9]. Certain cytokines, such as TNF-α, IL-6, IL-8, and TGF-β, have been detected at increased concentrations in MDS; this finding indicates that these cytokines participate in the dysplastic features of the hematopoietic cells in the bone marrow [10, 11].

Cytokines transmit signals that mediate inflammatory responses and regulate hematopoiesis by influencing the bone marrow microenvironment. Cytokines related to effector T cell function have not been investigated. IL-7 and IL-15 are members of the IL-2 receptor βγ-common chain (IL-2Rβγ_c_) family of cytokines. These cytokines play an essential role in the establishment and maintenance of normal immune function. As a T cell growth factor [12, 13], IL-15 signals through a complex containing a heterotrimeric high-affinity IL-15Rα chain and the IL-2Rβγ_c_. IL-15 is produced by numerous cell types in the context of an immune response, but primarily by members of the monocyte/macrophage lineage [14]. Given that myeloid cells are deregulated and the differentiation and function of T cells are modulated, we examined the level of IL-15 present in the peripheral blood of MDS patients. IL-15 is required for the optimal proliferation of CD8+ T cells and the homeostatic proliferation of CD8+ memory T cells [15, 16]. In the IL-2Rβγ_c_, IL-7 primarily maintains naïve T cells and provides survival-promoting extrinsic signals to T cells and B cells. IL-7 deficiency has been linked to severe combined immunodeficiency [17]. By contrast, the administration of IL-7 dramatically increases peripheral T cell numbers.

In this study, we analyzed the effect of IL-7 or IL-15 on T cell phenotype in MDS patients compared with healthy donors. Our data support a view in which the cytokine IL-15 could play an active role in phenotype conversion from naïve to terminal memory T cells.

## RESULTS

### Clinical characteristics of MDS patients

The characteristics of the MDS patients (*n* = 20) and healthy controls (*n* = 20) are summarized in Table 1. The mean age of controls was similar among controls (62.7 years, range 33–82 years) and MDS cases (63.5 years, range 35–83 years) (p = 0.17). Males and females were equally distributed in both study groups (p = 0.75). MDS patients were classified as refractory anemia with or without ringed sideroblast (*n* = 2, 10%), refractory cytopenia with multilineage dysplasia (*n* = 8, 40.0%), refractory anemia with excess blasts (RAEB)-1 (*n* = 3, 15%) and RAEB-2 (*n* = 4, 20%), and MDS-unclassified (*n* = 3, 20%) based on the classification criteria of the World Health Organization (WHO). Based on IPSS, seven patients (35.0%) were low risk, six patients (30%) were intermediate-1, four patients (20%) were intermediate-2, and three patients (15.0%) were high risk. Of 20 patients, 11 (55%) had identifiable cytogenetic abnormalities by metaphase karyotyping or FISH and 9 (45%) had normal cytogenetics.

**Table 1:** Clinical characteristics of MDS cases and controls

Characteristics of Cases and Controls			
**Age**			
Case: Controls	Mean age (range)		p-value
Controls (n=20)	62.7 (33-82)		
MDS Cases (n=20)	63.5 (35-83)		0.17
Sex (Male/Female)	N (M/F)	% (M/F)	
Controls (n=20)	10/10	50/50	
MDS Cases (n=20)	9/11	45/55	0.75
**Clinical Characteristics of MDS Cases**	n	%	
IPSS* classification			
Low	7	35	
Intermediate-1	6	30	
Intermediate-2	4	20	
High	3	15	
Cytogenetics			
Normal	9	45	
Abnormal	11	55	
WHO^##^ MDS subtype			
RA^#^ with ringed sideroblasts (RARS)	2	10	
RCMD^$^	8	40	
RA with excess blasts (RAEB)-1^%^	3	15	
RAEB-2	4	20	
MDS-unclassified (MDS-U)	3	15	

* IPSS=International Prognostic Scoring System;

## WHO=World Health Organization;

# RA=Refractory anemia

$ RCMD=Refractory cytopenia with multilineage dysplasia including patients with (n=1) or without ringed sideroblasts.

% Refractory anemia with excess blasts -1 and 2

### Increased IL-15 in MDS plasma

**Figure 1:**
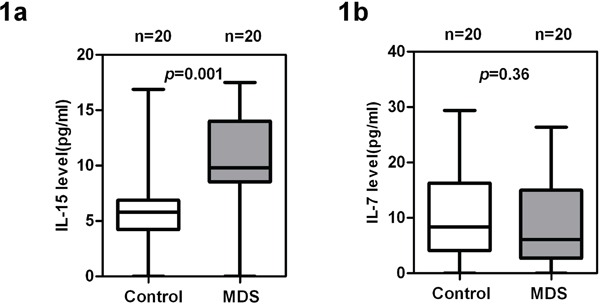
High levels of IL-15 and low levels of IL-7 in MDS patients compared with healthy donors Measurement of **a.** IL-15 and **b.** IL-7 levels in plasma of MDS patients (*n* = 20) and healthy controls (*n* = 20). IL-15 and IL-7 were analyzed in duplicate using the Luminex Performance Human High Sensitivity Cytokine Magnetic Panel B (R&D). Wilcoxon rank sum test was used for analysis. p values for the case and control differences are shown at the top of each panel.

We measured the IL-7 and IL-15 levels from the plasma of MDS patients and healthy controls. As shown in Figure 1a, IL-15 was significantly higher in MDS plasma [*n* = 20, median (25th–75th) percentile = 9.8 (8.55–13.75) pg/mL] than in healthy control plasma [*n* = 20, median (25th–75th) percentile = 5.8 (4.25–6.85) pg/mL, p = 0.001]. By contrast, IL-7 levels were similar among cases and controls (p = 0.36) (Figure 1b).

### Naïve T cell subset defects in CD4+ and CD8+ T cells in MDS

**Figure 2:**
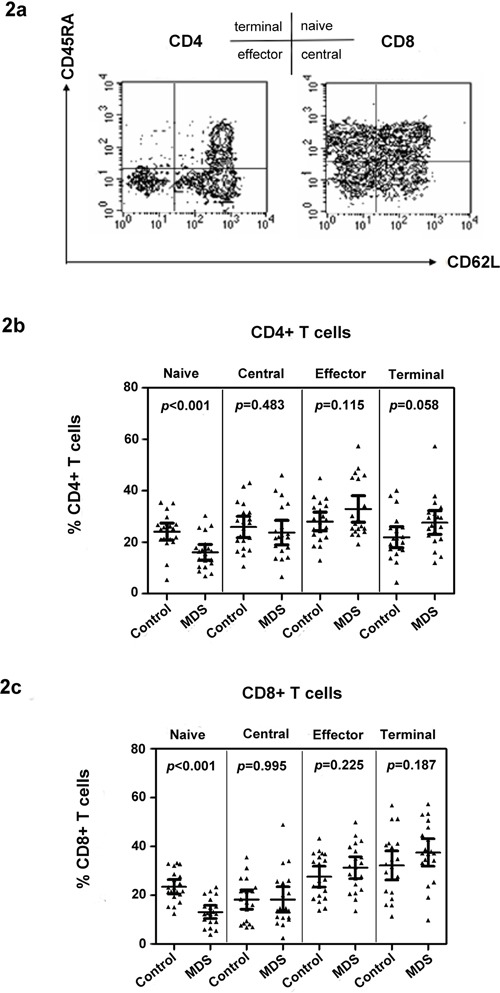
Naïve T cell subset defects in CD4+ and CD8+ T cells in MDS Examples of naïve and memory flow dot plots are shown using peripheral blood from MDS patients. Naïve and memory subpopulations were defined with antibodies to CD45RA and CD62L **a.** Case and control differences between CD4+ **b.** and CD8+ **c.** T cell subpopulations were compared in 20 controls and 20 MDS patients using the Wilcoxon rank sum test. p values for the case and control differences are shown at the top of each panel.

IL-15 is important in the differentiation of memory cells. Meanwhile, IL-7 supports the survival and expansion of naïve T cells. The phenotype of CD4+ and CD8+ T cells in MDS cases and controls was first examined by multicolor flow staining. CD45RA and CD62L were used to distinguish naïve and memory T cells [18], as defined previously and shown in Figure 2a. The proportion of circulating naïve and memory CD4+ and CD8+ T cell subpopulations was tested in MDS patients (*n* = 20) and age-matched healthy control donors (*n* = 20). Our data show that the percentage of naïve CD4+ and CD8+ T cells in MDS is significantly lower than that in healthy controls [16.11 ± 6.56 vs. 24.11 ± 7.18 for CD4+ T cell (p < 0.001); 13.15 ± 5.67 vs. 23.51 ± 6.25 for CD8+ T cell (p < 0.001)] (Figure 2b and 2c). Memory T cells can be divided into central memory, effector, and terminal memory based on the CD45RA and CD62L expression patterns. Effector and terminal memory CD4+ and CD8+ T cells were higher in MDS than in healthy controls, but the difference was insignificant for the two populations (Figure 2b and 2c).

### Correlation of IL-15 in plasma with naïve and effector memory T cells in MDS

**Figure 3:**
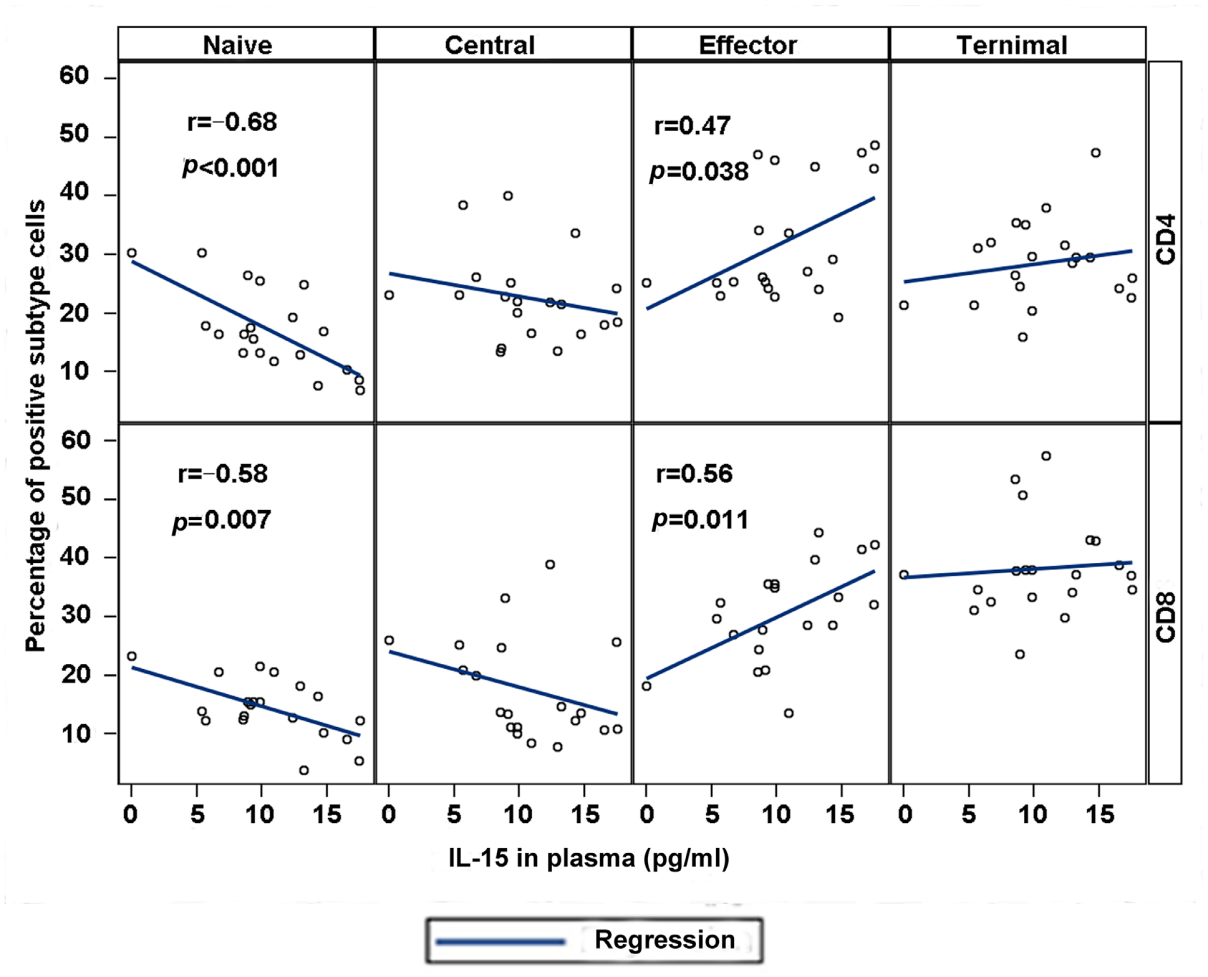
Plasma IL-15 correlates with phenotypic abnormalities in MDS Correlation between IL-15 plasma concentration and T cell subset percentages was conducted with correlation coefficients and p values for individual CD4 (top) and CD8 (bottom) panels. Highly significant correlations were observed between IL-15 and naïve cell population and effector T cells.

We conducted a correlation analysis between cytokines IL-15 and IL-7 and naïve and memory CD4+ or CD8+ T cells to investigate the possible relation of cytokines IL-15 and IL-7 to the phenotype of T cells. The correlation analysis indicated that the level of IL-15 in plasma is negatively associated with the percentage of naïve T cells in peripheral blood (*r* = −0.68, p < 0.001 for CD4+ naïve T cells; *r* = −0.58, p = 0.007 for CD8+ naïve T cells). By contrast, the level of IL-15 in plasma is positively correlated with the effector memory T cell percentage for CD4+ (*r* = 0.47, p = 0.038) and CD8+ (*r* = 0.56, p = 0.011) T cells (Figure 3). Central and terminal T cell percentage showed no correlation with IL-15, although a positive trend was observed for terminal CD8+ T cells (*r* = 0.18 for CD4+ T cells and *r* = 0.08 for CD8+ T cells, p > 0.05). However, no significant difference was observed between IL-7 level in plasma with naïve and memory CD4+ or CD8+ T cells in MDS patients.

### Effect of IL-7 and IL-15 treatment on T cell phenotype

**Figure 4:**
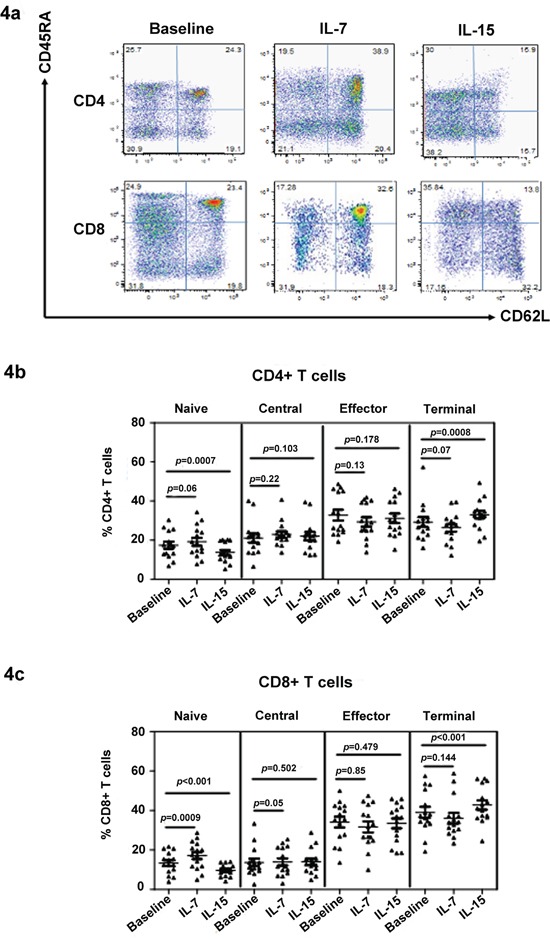
IL-7 restores the naïve T cell population in MDS The effect of IL-7 and IL-15 on T cell phenotype was determined by culturing the cell in these cytokines *in vitro*. A typical flow panel of the effect of IL-7 and IL-15 on T cell subset distribution in healthy controls is presented **a.** The naïve and memory percentage of CD4+ **b.** and CD8+ **c.** T cells with or without IL-7 and IL-15 treatment of PBMCs of MDS patients (*n* = 20). PBMCs were stimulated with IL-7 (10 ng/mL) or IL-15 (10 ng/mL) for 15 days. PBMC in the absence of cytokine was used as control. Medium was changed and cytokine was re-added every three days. At day 15, cells were collected for phenotype evaluation based on their naïve and memory T cell subtype distribution using CD45RA and CD62L staining. Wilcoxon signed-rank test was used for analysis. p values for the differences are shown at the top of each panel.

We also investigated the effect of IL-7 and IL-15 on T cell subset distribution using *in vitro* treatments. First, we tested the effect of IL-7 and IL-15 on T cells from healthy controls. As expected, IL-7 significantly increased the naïve cell proportion in culture after 15 days (Figure 4a). By contrast, IL-15 reduced the number of naïve cells. The reduction in naïve cells was associated with a corresponding increase in the percentage and total number of terminal memory cells, as shown in Figure 4a. Moreover, IL-15 was more important for CD8+ T cell survival because the CD8+/CD4+ ratio increased after IL-15 treatment (data not shown). Subsequently, peripheral blood mononuclear cells (PBMCs) from MDS patients were stimulated with IL-7 (10 ng/mL) and IL-15 (10 ng/mL) for 15 days (Figure 4b and 4c). To maintain survival in the control group, cells were pulsed with 20 ng/mL of IL-2 for 2 h on days 0, 5, and 10, followed by three washes. After IL-15 treatment, naïve CD4+ [17.45 ± 6.83 vs. 13.74 ± 4.99 (p < 0.0001)] and CD8+ [13.28 ± 5.56 vs. 9.65 ± 3.03 (p < 0.001)] T cells significantly decreased relative to the percentage at the baseline (Figure 4b and 4c). Meanwhile, CD45RA+/CD62L− terminal memory T cells significantly increased in CD4+ [29.24 ± 9.68 vs. 33.00 ± 7.66 (p = 0.008)] and CD8+ [38.98 ± 10.87 vs. 42.81 ± 9.26 (p < 0.001)] subpopulations compared with the baseline group. After IL-7 treatment in MDS patients, naïve cells significantly increased in CD4+ [17.45 ± 6.83 vs. 20.17 ± 7.13 (p < 0.05) and CD8+ [13.28± 5.56 vs. 17.13 ± 7.02 (p < 0.001)] subpopulations compared with the baseline group. In addition, a trend of lower terminal memory and higher central memory CD8+ T cells (p = 0.07 and p = 0.05, respectively) was observed after IL-7 treatment (Figure 4c). Although an increase in naïve cells was observed, this increase can be considered partial restoration relative to healthy donors. During 15 days of cell culture *in vitro*, no significant change in total cell number was observed.

## DISCUSSION

MDS is a clinically and molecularly heterogeneous group of clonal hematopoietic stem cell disorders that are singularly characterized by peripheral blood cytopenias from ineffective hematopoiesis and an increased but variable risk of leukemic transformation [19, 20]. Evidence is accumulating that cellular immune dysfunction and aberrant cytokine expression are important in MDS pathogenesis. Failure of the bone marrow to produce healthy cells is a gradual process in MDS; its circulating blood cells may not function properly because of dysplasia, and several patients will progress to acute AML. Cytokines within the bone marrow microenvironment play a role in disease development and prognosis [21–24]. IL-7 and IL-15 are critical regulators of T cell and NK cell proliferation and differentiation. Although these pivotal cytokines support both adaptive and innate immune responses, no studies have related MDS-associated T cell abnormalities to these cytokines [25–28]. Our present study investigates the effect of IL-7 and IL-15 on T cell phenotype in MDS.

Various aspects of immune deregulation were observed in MDS patients, such as reduced CD4/CD8 ratio [7] and diminished CD4+ and CD8+ T cell receptor restriction. Cytokines are used by body to transmit signals related to immune system issues between cells, mediate inflammatory responses and regulate hematopoiesis by influencing bone marrow microenvironment. IL-7 and IL-15, the members of common gamma-chain family cytokine, are essential for establishment of maintenance of normal immune system function. As a T cell growth factors [12, 13], IL-15 plays an important role in the development and differentiation of T cell and NK cells, it is required for the proliferation of CD8+ T cells [24, 26, 27]. IL-7 can maintain human T cell development and provides survival-promoting extrinsic signal to T cells and B cells, it has been shown to be critical for lymphocyte homeostasis. Cytokines IL-7 and IL-15 play a critical role in maintaining the naïve and memory subpopulations. We examined cytokines IL-7 and IL-15 for T cell phenotype conversion evaluation to explore the effect of IL-7 and IL-15 on the phenotype of T cells of MDS. IL-7 can maintain the naïve subtype at a relative high level, whereas IL-15 can decrease the naïve subtype and increase the terminal memory cell. IL-7 is more important for CD4+ T cells to survive and proliferate. CD4+/CD8+ T cell ratio could be kept at a relatively high level in cultured PBMC in the presence of the IL-7 group. For the IL-15-treated PBMCs, the condition of CD4+ T cells is not as good as that in the IL-7-treated group; specifically, its CD4+/CD8+ T cell ratio dramatically decreases. Decreased CD4+/CD8+ T cell ratio and a low proportion of naïve/higher effector memory T cells are the characteristics of MDS patients. Similar features are demonstrated in our study in which PBMCs were stimulated with IL-15.

Accurately distinguishing naive T cells is particularly important, the use of any single marker, for example CD45RA or CD45RO, is poor to identify naive cells. In the past, a single marker, such as expression of CD45RA or lack of expression of CD45RO, has been used to evaluate naive cell counts, but that usually leads to discordant T cell sub-population. In present study, we applied multiple markers to improve the specificity for naive and memory cells in MDS patients.

The naïve and memory cell populations are regulated in different ways within the T-cell pool. IL-7 play an important role in proliferation and differentiation of naïve T cells by promoting expression of Bcl-2, its importance in promoting survival of naïve T cells was demonstrated in adoptive transfer models, in which IL-7^+/+^ cells disappeared rapidly after transfer into IL-7^−/−^ mice [23]. Naïve T cells could undergo homeostatic proliferation in IL-4^−^ and IL-15^−^ hosts, but can proliferate only minimally in IL7^−^ hosts. Except for homeostatic proliferation, the extended survival of naïve T cells requires IL-7, otherwise naïve T cells will gradually disappeared [13]. In contrast to IL-7, IL-15 stimulates proliferation of memory T cells but not naïve-phenotype (CD44^lo^) CD8^+^ T cells *in vivo* and *in vitro* [29], both the number and proliferation rate of CD8 memory cells could be reduced in IL-15^−/−^ and IL-15Rα^−/−^ mice model.

We noticed that IL-7 can increase naïve T cell number during *in vitro* experiments. However, although there is no significant difference of plasma IL-7 between controls and MDS patients, a lower naïve proportion is demonstrated in MDS patients compared to healthy controls. Therefore, we tested the phenotype alteration of CD4+ and CD8+ T cells in the presence of IL-7 and IL-15 (data is not shown), it is displayed that the terminal cells increased and naïve subtype decrease when PBMC were treated with both IL-7 and IL-15 simultaneously, the effect of IL-7 was overlapped by IL-15, which may partly explain why a lower naïve T cell was detected in MDS patients although no abnormal plasma IL-7 level is observed.

Phenotype conversion could happen due to CD62L cleavage, for example, CD62L will be rapidly shed and naïve phenotype will disappear once the T cells have been activated with PMA/ionomycin. However, the phenotype change by IL-15 is not by such way. After having investigated phenotype alteration at different time points, we found that part of naïve T cells developed to central memory cells at early time, afterwards, central memory cells further convert to effector and terminal cells, which suggests that the phenotype conversion by IL-15 followed a linear model: from naïve, central memory, effector memory to terminal memory.

IL-15 is a pro-inflammatory cytokine and produced primarily by dendritic cells, monocytes, and epithelial cells. Abnormality of IL-15 expression has been detected in patients with autoimmune diseases and lymphoid malignancies. IL-15 overexpression can initiate leukemic transformation in murine models and that malignant transformation of both NK- and T-cells can develop [30]. In present study, we observed a higher plasma IL-15 level in MDS patients; meanwhile, a lower naïve and higher terminal memory subtype T cells were demonstrated. It is not clear how increased plasma IL-15 level happened in MDS, but quite likely that an immune activation is associated with MDS, which could lead to alteration of IL-15 product. Conversely, increased IL-15 may drive progress of MDS. Our recent analysis indicated that naïve CD8+ T cell is very low and terminal CD8+ T cells very high in LGL leukemia (data not shown), such cytokine alteration and T cell phenotype pattern are quite similar as that MDS does, such type of MDS patients may have a worse body's ability to fight infections and be more inclined to transform to AML. Whether elevated IL-15 involved in the pathogenesis of MDS remains to be further characterized.

Reduction in naïve cells in MDS may be mediated by either increased apoptotic sensitivity or increased rate of phenotype conversion to memory cells [31, 32]. Our results implicate IL-15 as a contributor to changes in the percentage of naïve CD4+ and CD8+ T cells. Moreover, a relatively higher IL-15 level was detected in MDS plasma compared with healthy control plasma in the current study. IL-15 plasma levels not only negatively correlated with naïve T cell percentage but also converted naïve cells to memory cells *in vitro* in the healthy donors and MDS patients. In addition, our results indicate that the addition of IL-7 may stabilize or recover naïve T cell populations, probably improving effector immune responses.

In summary, given that little is known about the effect of IL-7 and IL-15 on the phenotype of T cells in MDS patients, the current study investigated the possible mechanism of T cell dysfunction in patients with MDS. We reported the effect of IL-7 and IL-15 on T cell phenotype in MDS patients. Our findings provide important insight into the mechanism of cellular immune dysfunction in MDS patients and indicate the possible role of IL-15 in T cell phenotype conversion. IL-7 may be a possible option to maintain naïve T cell subpopulation at a relatively high level.

## MATERIALS AND METHODS

### Patients and healthy controls

The present study comprised 20 consecutive patients with primary MDS and 20 age-matched healthy controls. The characteristics of the patients are shown in Table 1. MDS patients were recruited from the Tianjin Cancer Hospital and Tangshan Hospital. Diagnoses were made according to the WHO criteria. All patients were clinically stable and untreated when the sample was collected. After a written informed consent was obtained, peripheral blood was collected in heparinized tubes from each patient and healthy control. Healthy controls (*n* = 20) were obtained from healthy volunteers in our hospital. For plasma collection, blood was centrifuged for 15 min at 1,000×*g* using a refrigerated centrifuge and stored at −80°C. PBMCs were isolated by Ficoll-Hypaque density gradient centrifugation (Amersham Pharmacia Biotech, Piscataway, NJ, USA) following the protocol of the manufacturer. These cells were also freshly used for *in vitro* cytokine intervention experiment or were frozen in liquid nitrogen.

### IL-7 and IL-15 in MDS and healthy controls

Plasma collected from peripheral blood was used for cytokine analysis. The level of IL-7 and IL-15 in plasma was analyzed in duplicate using Luminex Performance Human High Sensitivity Cytokine Magnetic Panel B (R&D Systems, Inc., Minneapolis, MN, USA). Briefly, IL-7-specific and IL-15-specific antibodies were pre-coated on color-coded magnetic microparticles. Standards were used for quantification, and microparticles without antibody were used for background analysis. All samples were added to 96-well plates, followed by washing to remove unbound substances. Then, streptavidin–phycoerythrin conjugate was added to the wells. Following washing, microparticles were resuspended and read by the Luminex100 MAGPIX Analyzer. Data were evaluated with Xponent software (Luminex Corporation, Austin, TX, USA). The observed intensities of duplicate samples were averaged and mapped to a fitted standard curve created from a serial dilution series of known cytokine. Detected intensities below the standard range were marked as 0%.

### Detection of naïve and memory T cell subpopulations

Cryopreserved PBMCs were thawed and incubated with antibodies to distinguish several cellular populations based on their phenotype. Briefly, CD4 and CD8 T cells were labeled with anti-CD3-phycoerythrin Cy7, anti-CD45RA-FITC, anti-CD62L-APC, and either anti-CD4-APC Cy7 or anti-CD8-APC Cy7. All antibodies were obtained from BD Biosciences (San Jose, CA USA). Isoforms of the tyrosine phosphatase CD45RA and l-selectin (CD62L) have been used to distinguish naïve T cells from memory T cells [15]. 4′, 6-diamindigo-Z-phenylinodole (DAPI) was used to distinguish cell viability. The results of flow cytometry were analyzed on an LSRII Benchtop Analyzer (BD Bioscience, San Jose, CA, USA).

### Measurement of T cell phenotype after cytokine treatment with IL-7 or IL-15

PBMCs were stimulated with IL-7 (10 ng/mL) or IL-15 (10 ng/mL) (eBioscience, San Diego, CA, USA) for 15 days to investigate the possible effect of cytokines IL-7 and IL-15 on the phenotype of T cells. PBMC in the absence of cytokine IL-7/IL-15 was used as control. Medium was changed and cytokine was re-added every three days. To ensure that the cells survived until the end of the experiment, PBMCs of the control group were pulsed with 20 ng/mL of IL-2 for 2 h at days 5 and 10, followed by washing thrice. At day 15, cells were collected for phenotype evaluation based on their naïve and memory T cell subtype distribution.

### Statistical analysis

Wilcoxon rank sum test was conducted for independent samples with ordinal distribution. Wilcoxon signed-rank test was used for the comparison of matched pair samples without normal distribution. The *t* test was also applied to compare the mean difference for independent samples with normal distribution. The paired *t* test was used to compare phenotype difference between without and with cytokine treatment. All statistical analyses were conducted with SAS 9.2. Differences were considered significant when the two-tailed p value was less than 0.05.
